# Mandibular orthopedic advancement in different facial patterns and distinct stages of skeletal maturation

**DOI:** 10.1590/2177-6709.26.2.e21bbo2

**Published:** 2021-05-17

**Authors:** Fernando Penteado Lopes da SILVA

**Affiliations:** 1 Diplomate of the Brazilian Board of Orthodontics and Facial Orthopedics.; 2 Private practice (Campinas/SP, Brazil).

**Keywords:** Herbst appliance, Class II orthopedic treatment, Mandibular retrognathism, Skeletal maturation

## Abstract

The Herbst appliance can be very effective in treatment of Class II patients with mandibular retrognathism. Because of the continuous action in a full-time basis, treatment time using it normally takes from six to ten months, and is usually followed by a second phase of full fixed appliances, in order to obtain both occlusal refinement and long term stability. Despite Herbst appliance’s effectiveness in the occlusal and dentoalveolar perspectives, its facial results may differ among patients with different growth patterns, as well as in distinct stages of skeletal maturation. In the current paper, two patients with different facial patterns are presented, who were treated under the same protocol, using Herbst and full fixed appliances in different skeletal maturation stages, and both dentoalveolar and facial results are compared and discussed.

## RESUMO

Um número significativo de pacientes que procuram o tratamento ortodôntico apresenta má oclusão de Classe II acompanhada pelo retrognatismo mandibular. Abordagens ortopédicas para avanço mandibular são comumente utilizadas enquanto houver crescimento facial remanescente e, nesses casos, o estágio de maturação esquelética deve ser avaliado para definir a melhor época de intervenção terapêutica. Após concluída a fase ortopédica, normalmente é realizada uma segunda fase ortodôntica para refinamento oclusal, com o intuito de oferecer maior estabilidade das correções em longo prazo. No presente artigo, serão discutidos os resultados do avanço mandibular ortopédico considerando-se diferentes estágios de crescimento.

## INTRODUCTION

The negative effect caused by mandibular retrognathism on the face is often the reason why adult patients seek orthodontic-surgical treatment approaches.^1^ ^-^ ^5^ Patients treated during craniofacial growth stages, on the other hand, may have benefits from the orthopedic mandibular advancement. The use of fixed as well as removable orthopedic appliances moves the mandible forward, in order to correct the initial sagittal discrepancy.^6^

The Herbst appliance is probably the most used fixed orthopedic device, which uses intermaxillary anchorage, by means of a telescopic mechanism, to promote orthopedic mandibular advancement. Brought back to the orthodontic literature by Pancherz, it combines both orthodontic and orthopedic effects during the correction of mandibular retrognathism, and one of its main advantages is inducing continuous mandibular advancement during rest and masticatory function.^6^ ^-^ ^10^ The Herbst appliance has been studied for several years, and both dental and skeletal effects have been widely advocated.^11^ ^-^ ^13^ Among these effects, the dental compensations must be highlighted, since they are present after treatment with both fixed and removable orthopedic devices.^14^ ^,^ ^15^ Located mainly in mandibular anterior^16^ ^-^ ^20^ and maxillary posterior^21^ dentoalveolar regions, dental compensations play a fundamental role during orthopedic mandibular advancement in Class II patients’ treatment. 

After the orthopedic correction, a second orthodontic treatment phase is necessary, in order to obtain adjustments such as improvement of dental crowding, closing residual spaces and occlusal refinement.^22^ It is widely known that a stable occlusal intercuspation obtained after mandibular advancement plays an essential role in the long term stability.^23^

However, the ideal period of orthopedic mandibular advancement treatment, using either fixed or removable devices, still remains a controversial issue among authors. Depending on the growth stage, the treatment is considered as early approach if started during deciduous or early mixed dentition, or before pubertal growth spurt; on the other hand, it is considered as a late approach if started during late mixed or permanent dentition, or during or after pubertal growth spurt. Considering this context, in which of these periods would be appropriate to start orthopedic mandibular advancement? In case of treatment during effective pubertal growth period, what would be its repercussions in the long term, and there would be stability warranties? Searching for answers to these questions, a meta-analysis was performed to evaluate if the treatment onset time would bring any difference in effects of mandibular orthopedic advancement in patients during distinct growth stages.^24^ In patients treated before pubertal growth spurt, the mandibular length increased from 0.89 to 1.68mm (mean value = 1.29mm), while those patients treated during pubertal growth spurt presented mandibular length increasing from 3.65 to 5.00mm (mean value = 4.32mm). The authors pointed out that mandibular growth can be effectively augmented only if orthopedic advancement is performed during pubertal growth periods, and also that the ideal period should be defined using appropriate methods, such as hand-wrist radiographs. 

The ideal treatment period, however, is not determined solely by biological parameters. The psychosocial aspect of the patient must be considered, as well as the risk of trauma to the maxillary incisors, which is also very common in Class II patients with mandibular retrognathism. In a recent systematic review with 27 randomized clinical trials and a total of 1,251 patients, groups underwent early or late orthopedic treatment, and also no treatment samples were compared. In the comparison between early and late treatments, the only significant difference was a decreased incidence of maxillary incisor trauma, which was also found in the comparison between late treatment and no treatment groups.^25^

In the present paper, two case reports will be presented, of Class II patients treated in distinct stages of skeletal maturation, using the same protocol of mandibular orthopedic advancement, followed by a full fixed appliances orthodontic treatment.

### CASE 1

#### DIAGNOSIS

**Figure 1:**
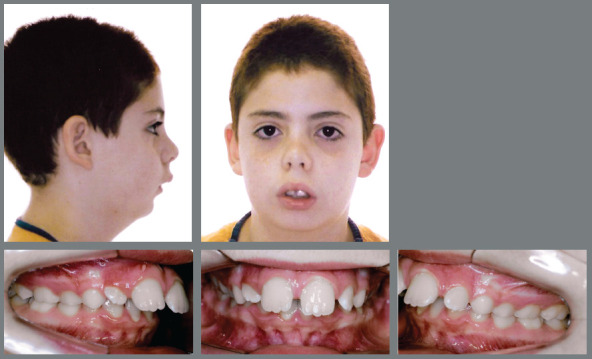
Initial extraoral and intraoral photographs.

**Figure 2:**
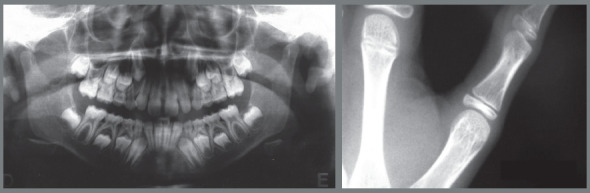
Initial panoramic and hand-wrist radiographs.

**Figure 3:**
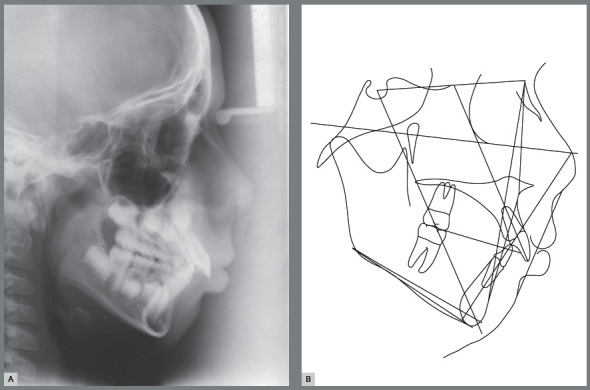
Initial cephalometric radiograph of facial profile **(**A) and cephalometric tracing **(**B).

The case 1 refers to a young male patient, at 7 years and 10 months of age, with the following chief complaints: *“excessive spaces between upper frontal teeth”* and *“chin in a backward position”,* as well as some respiratory difficulty and introspective social behavior. According to the facial analysis, the mandibular retrognathism was evident, as well as the labial incompetence and a hiperdivergent growth pattern, associated with the increased lower facial height and a clockwise mandibular rotation. Despite the good position of the upper lip, the nasolabial angle was augmented. The lower lip was in a backward position and slightly everted (Fig 1). The patient had an Angle Class II division 1 malocclusion during the intermediate period of mixed dentition, associated with increased overbite and overjet (Figs 1 and 2). The hand-wrist radiograph highlighted the growth stage of the patient, which was before pubertal spurt (Fig 2). Cephalometric analysis was in accordance with facial remarks, which indicated a hyperdivergent facial growth pattern (FMA = 27.1^o^, SN.GoGn = 33.5^o^ and Y-axis = 69.5^o^). It was also detected a slight maxillary protrusion (SNA = 83.2^o^) and mandibular retrusion (SNB=76.9^o^), which led to an increased skeletal profile convexity (ANB = 6.3^o^ and Convexity Angle = 12.9^o^) (Tab. 1). The maxillary incisors were protruded and positioned in a buccal position (1.NA = 25.9^o^ and 1-NA = 5.5mm), while the mandibular incisors were relatively well positioned in mandibular apical base (IMPA=89.9^o^, 1.NB = 23.9^o^ and 1-NB = 6.0mm) (Fig 3, Tab. 1).

### TREATMENT PLAN AND PROGRESS

Considering the functional and psychosocial issues of patient, the orthopedic mandibular advancement approach was promptly accepted by his parents. It was also explained that an orthodontic-surgical approach could be necessary in the future, in case of a unsuccessful orthopedic treatment. In these terms, the mandibular orthopedic advancement was initiated with a Herbst appliance designed for mixed dentition.^27^ After the accomplishment of orthopedic approach (Phase 1), an orthodontic stage with full fixed appliances took place (Phase 2), in order to obtain occlusal refinement. 

**Figure 4:**
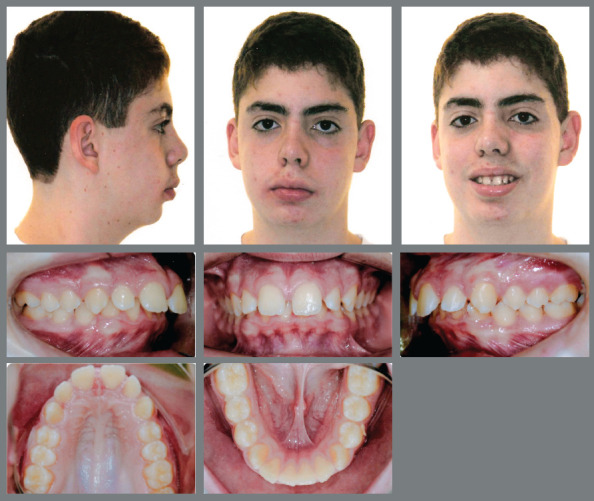
Intermediate extraoral and intraoral photographs after Phase 1.

**Figure 5:**
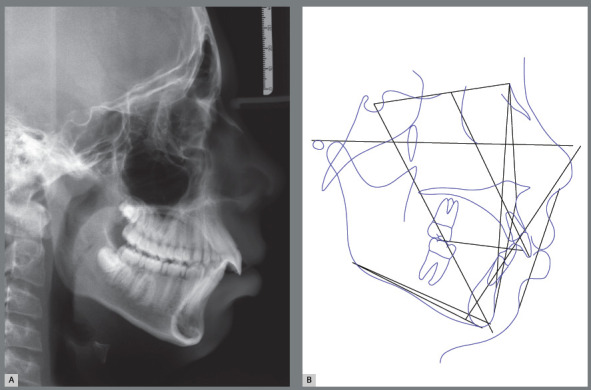
Intermediate cephalometric radiograph of facial profile **(**A) and cephalometric tracing **(**B) after Phase 1.

The Class II malocclusion with mandibular retrognathism is usually accompanied by a narrow maxilla,^3^ ^,^ ^6^ ^,^ ^27^ and these features were also present in this case. Thus, a Rapid Maxillary Expansion (RME) was previously performed with a Haas expander, in order to adequate the maxilla before the mandibular advancement. The Herbst appliance was kept in place continually during a one-year period, and then removed when both adequate overjet and overbite were achieved. New orthodontic records were taken in permanent dentition stage, as soon as second molars erupted (Figs 4 and 5).

**Figure 6:**
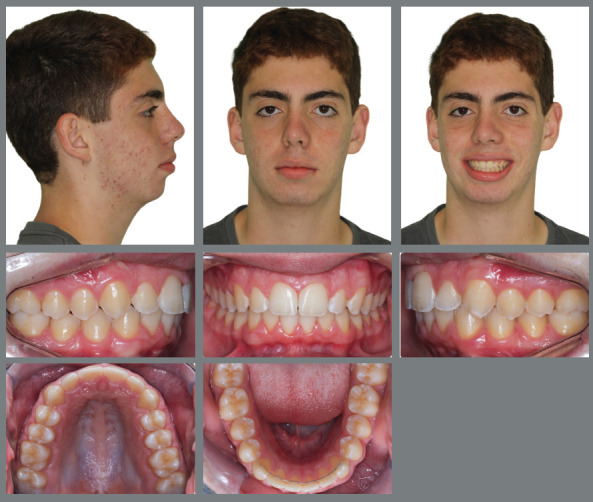
Final extraoral and intraoral photographs.

**Figure 7:**
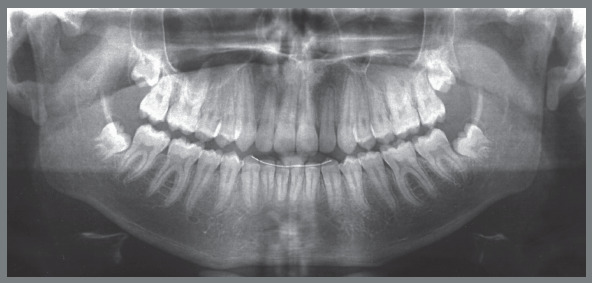
Final panoramic radiograph.

**Figure 8:**
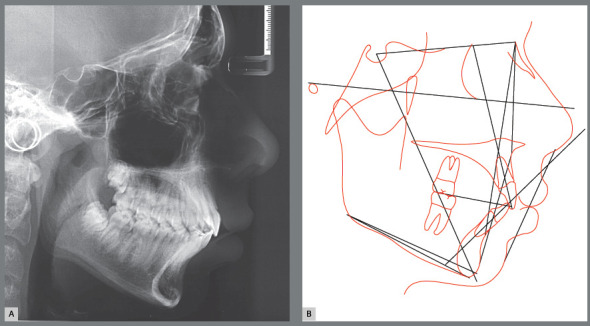
Final cephalometric radiograph of facial profile **(**A) and cephalometric tracing **(**B).

During the full fixed orthodontic appliances stage (Phase 2), which took place during a period of 18 months, the remaining spaces were closed and correction of the overbite was improved, as well as the maintenance of dental compensations obtained during Phase 1. The following retention period was performed with an upper Hawley retainer and a lower fixed canine-to-canine lingual bar. In the final records, it is possible to observe the Class I dental relation, as well as the correct overjet and overbite (Figs 6, 7 and 8).

**Figure 9:**
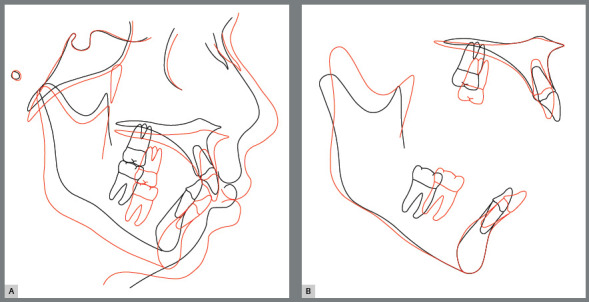
Total **(A)** and partial **(B)** superimpositions of cephalometric tracings at start (black line) and end (red line) of treatment.

The pre- and post-treatment cephalometric tracings superimposition highlighted a clockwise mandibular rotation, with fulcrum at the condylar region. The maxillomandibular structures were actually moved to the same direction, as a consequence of the hyperdivergent facial growth pattern (Fig 9). 

## CASE 2

### DIAGNOSIS

**Figure 10:**
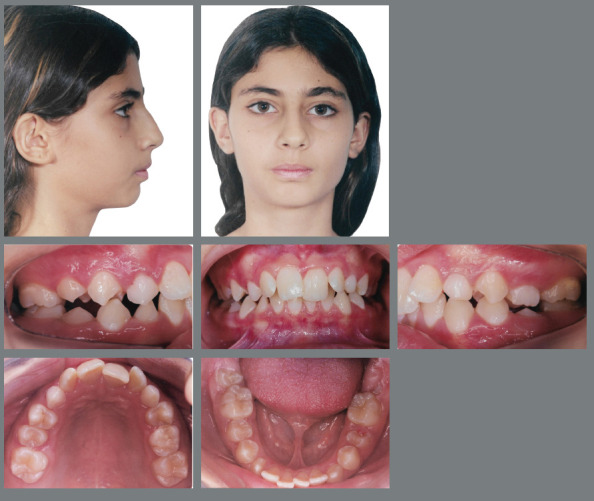
Initial extraoral and intraoral photographs.

**Figure 11:**
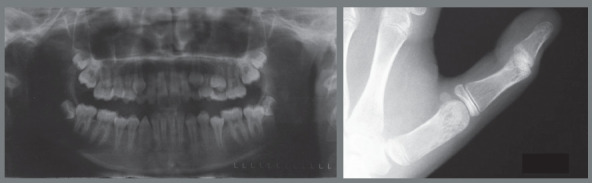
Initial panoramic and hand-wrist radiographs.

**Figure 12:**
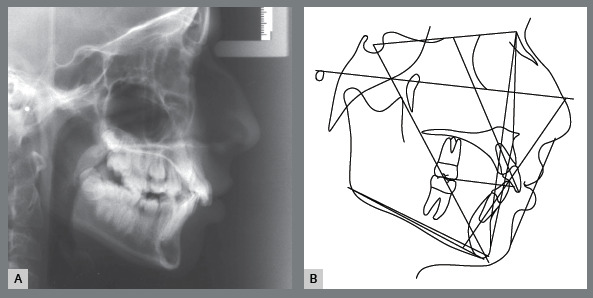
Initial cephalometric radiograph of facial profile **(**A) and cephalometric tracing **(B**).

Female patient, with 11 years and 10 months of age, sought orthodontic treatment with chief complaints related to maxillary anterior teeth crowding, as well as unaesthetic facial characteristics. The facial analysis highlighted a maxillomandibular retrusion associated to both mandibular retrognathism and increased nasolabial angle.^3^ The patient presented a Class II, division 2 malocclusion at the final stage of mixed dentition, with maxillary and mandibular incisor crowding, and good labial competence (Fig 10). The hand-wrist radiograph evaluation highlighted the presence of sesamoid bone of the thumb with adequate radiopacity, as well as epiphyseal covering stage, and pointed to the peak of pubertal growth spurt (Fig 11). The cephalometric analysis indicated a good facial balance and normal growth pattern (FMA = 21.8^o^, SN.GoGn = 31.5^o^, Y-axis = 66.9^o^). The Class II malocclusion was correlated to the mandibular retrognathism (ANB = 6.5^o^, SNB = 76.5^o^, SNA = 83^o^) and to the skeletal facial profile convexity (angle of convexity = 10.0^o^) (Fig 12,Tab. 2). The maxillary incisors were well positioned (1.NA = 24.9^o^, 1-NA = 4.1mm), while the mandibular incisors were slightly proclined (IMPA = 96.7^o^, 1.NB = 26.9^o^, 1-NB = 5.6mm) (Fig 12, Tab. 2). 

### TREATMENT PLAN AND PROGRESS

**Figure 13:**
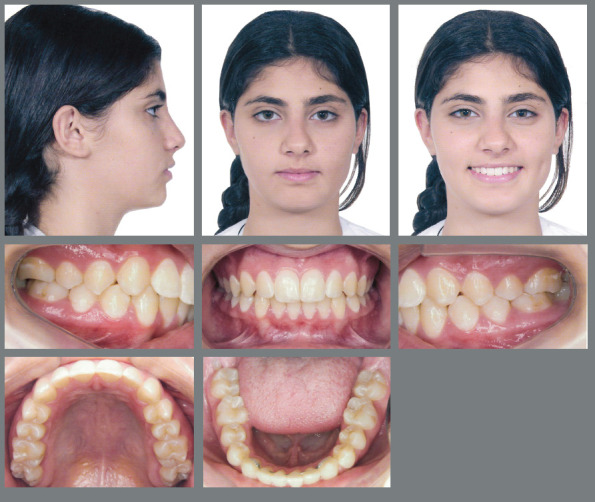
Final extraoral and intraoral photographs.

**Figure 14:**
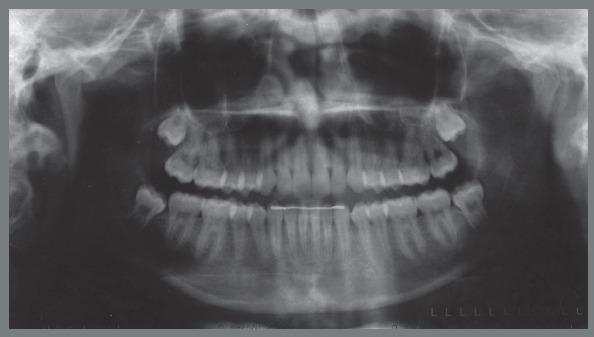
Final panoramic radiograph.

**Figure 15:**
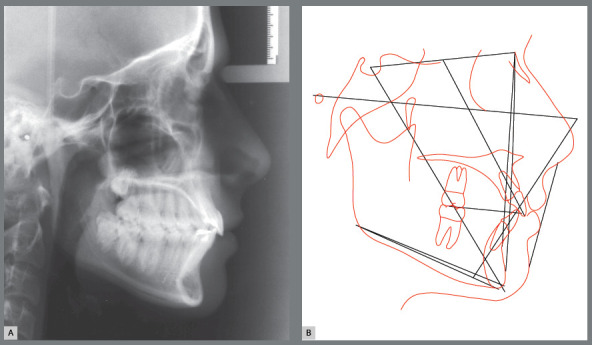
Final cephalometric radiograph of facial profile **(**A) and cephalometric tracing **(**B).

The mandatory decompensations on maxillary dental arch were performed before the mandibular orthopedic advancement. Thus, Rapid Maxillary Expansion (RME) followed by upper 4x2 alignment and leveling were performed. Then, orthopedic mandibular advancement was performed with Herbst appliance, which was kept in place for a period of 10 months. After the orthopedic phase was accomplished, a full orthodontic appliances stage took place, in order to obtain occlusal refinement, for a period of 18 months. During the retention phase, the patient worn an upper Hawley and a lingual lower canine-to-canine fixed bar (Figs 13, 14 and 15).

**Figure 16:**
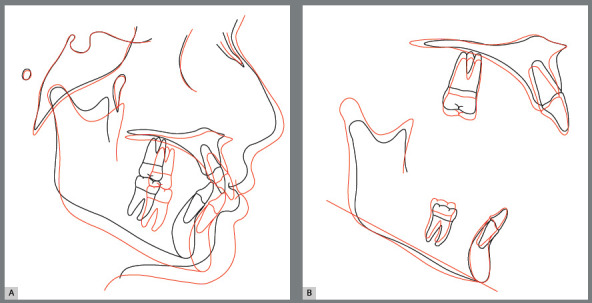
Total **(A)** and partial **(B)** superimpositions of cephalometric tracings at start (black line) and end (red line) of treatment.

Along the decrease of mentolabial angle, there was also an improvement of the relationship between the upper and lower lips. Regarding the maxillary incisors, there was a slight proclination as a consequence of their long-axis correction. The maxillary cephalometric tracings superimposition evidenced a slight backward movement of point A, which probably happened as a consequence of remodeling on this region that was also depicted by decrease of SNA in the post-treatment. The maxillary molars presented slight extrusion and remained at the same anteroposterior positions. There was also extrusion movement of mandibular molars, as a consequence of the vertical dentoalveolar remodeling caused by mandibular advancement with Herbst appliance^6,7,9^ (Fig 16).

## DISCUSSION

In both cases, there was improvement of facial characteristics at the end of treatment, mainly due to the achievement of spontaneous lip contact at rest in Case 1, as well as decrease of mentolabial sulcus in Case 2. The decrease of facial profile convexity also took place in both cases at the end of treatment, and it was more remarkable in Case 2. The hyperdivergent facial pattern of Case 1 led to a more vertical than sagittal movement of the mandible. The evaluation of Table 1 highlights that cephalometric measures regarding to skeletal facial pattern increased, as a consequence of active facial growth during the period of orthopedic mandibular advancement. 

**Table 1:** Cephalometric values at start **(A)** and at the end **(C)** of treatment.

	MEASURES		Normal	A	B	C	≠ A/C
Skeletal pattern	SNA	(Steiner)	82°	83.2°	84.5°	82.8°	0.4
	SNB	(Steiner)	80°	76.9°	77.4°	75.9°	1.0
	ANB	(Steiner)	2°	6.3°	7.1°	6.9°	0.6
	Wits	(Jacobson)	♀ 0 ±2mm ♂ 1 ±2mm	0.3mm	4.4mm	5.8mm	5.5
	Angle of convexity	(Downs)	0°	12.9°	14.4°	14.0°	1.1
	Y-Axis	(Downs)	59°	69.5°	71.1°	70.7°	1.2
	Facial Angle	(Downs)	87°	86.7°	87.3°	86.2°	0.5
	SN.GoGn	(Steiner)	32°	33.5°	32.9°	29.4°	4.1
	FMA	(Tweed)	25°	27.1°	25.7°	22.1°	5.0
Dental pattern	IMPA	(Tweed)	90°	89.9°	96.7°	108.6°	18.7
	1.NA (degrees)	(Steiner)	22°	25.9°	20.5°	15.6°	10.3
	1-NA (mm)	(Steiner)	4 mm	5.5mm	5.8mm	3.0mm	2.5
	1.NB (degrees)	(Steiner)	25°	23.9°	29.8°	37.0°	13.1
	1-NB (mm)	(Steiner)	4mm	6.0mm	7.7mm	8.2mm	2.2
	- Interincisal angle	(Downs)	130°	123.9°	122.6°	120.4°	3.5
	1 - APg	(Ricketts)	1mm	12.5mm	12.3mm	8.5mm	4.0
Profile	Upper Lip - Line S	(Steiner)	0mm	5.3mm	4.6mm	4.6mm	0.7
	Lower Lip - Line S	(Steiner)	0mm	7.8mm	7.7mm	7.2mm	0.6

**Table 2:** Cephalometric values at start **(A)** and at the end **(B)** of treatment.

	MEASURES		Normal	A	B	≠ A/B
Skeletal pattern	SNA	(Steiner)	82°	83°	82.6°	0.4
	SNB	(Steiner)	80°	76.5°	80.2°	3.7
	ANB	(Steiner)	2°	6.5°	2.4°	4.1
	Wits	(Jacobson)	♀ 0 ±2mm ♂ 1 ±2mm	6.0mm	1.3mm	4.7
	Angle of convexity	(Downs)	0°	10.0°	2.4°	7.6
	Y-Axis	(Downs)	59°	66.9°	64.8°	2.1
	Facial Angle	(Downs)	87°	89.3°	92.6°	3.3
	SN.GoGn	(Steiner)	32°	31.5°	28.3°	3.2
	FMA	(Tweed)	25°	21.8°	19.0°	2.8
Dental pattern	IMPA	(Tweed)	90°	96.7°	99°	2.3
	1.NA (degrees)	(Steiner)	22°	24.9°	28.9°	4.0
	1-NA (mm)	(Steiner)	4 mm	4.1mm	6.8mm	2.7
	1.NB (degrees)	(Steiner)	25°	26.9°	29.2°	2.3
	1-NB (mm)	(Steiner)	4mm	5.6mm	6.2mm	0.6
	- Interincisal angle	(Downs)	130°	122.4°	119.4°	3.0
	1 - APg	(Ricketts)	1mm	7.7mm	7.5mm	0.2
Profile	Upper Lip - Line S	(Steiner)	0mm	0.4mm	-0.9mm	1.3
	Lower Lip - Line S	(Steiner)	0mm	3.0mm	0.0mm	3.0

The comparison of Herbst effects in different facial patterns has been studied for several years, and often presents similar results regarding to the maintenance of mandibular growth direction, as well as to a satisfactory dentoalveolar correction, as recently demonstrated by Atresh et al.^28^ Regarding the characteristics of the initial malocclusions of both cases in this current paper, Class II division 1 (Case 1) and Class II division 2 (Case 2), distinct skeletal results after Herbst therapy were observed, mainly related to a more effective forward mandibular movement in the hypodivergent patient (Case 2), which was at the peak of pubertal growth spurt. 

Despite some advantages, compared to other devices, specially regarding the immediate facial positive change, as well as to the full-time mandibular forward position during its use, it is reasonable to admit some limitations of Herbst appliance. The tooth-tissue-borne anchorage promotes significant dental compensation effects, as can be observed in Case 1. Furthermore, its action on the mandibular growth is only temporary.^26^ In both of the presented cases, the orthopedic mandibular advancement was maintained during a one-year period in a full-time basis, as suggested by Pancherz.^29^ ^,^ ^30^

The SNB angle presented a slight increase of 0.5^o^ in Case 1 after Herbst removal, and decreased to 1.5^o^ at the end of full fixed appliances phase (Table 1). The angle of facial convexity increased after the mandibular advancement, and remained the same after the full fixed appliances phase. The same tendency was observed regarding the ANB angle. Considering vertical dimension, the FMA and SN.GoGn angles decreased, suggesting a slight improvement on facial growth direction. Regarding the Y-axis, however, significant changes were not observed. The possibility of inducing real additional mandibular growth is still far from being unanimity in the literature, and the amount of orthopedic effect depends mainly on facial growth pattern, as well as on the skeletal age at treatment onset.^6^ ^,^ ^30^

Bearing in mind the negative psychosocial factors present in Case 1, which were caused mainly by the mandibular retrognathism and facial characteristics, it was decided to start treatment during early mixed dentition period. This choice was made in order to improve the facial appearance of the patient as well as his self-esteem. Besides, it reduces the occurrence of trauma to the maxillary incisors.^25^ ^,^ ^31^ The disadvantage of the early approach, however, relies on the long total treatment time and, usually, a less effective mandibular response to the orthopedic advancement. The active period of Herbst appliance in Case 1 lasted 10 months, which is in accordance to the recommended in the literature.^23^ ^,^ ^26^ ^,^ ^27^

In contrast to the minor skeletal changes, the dentoalveolar cephalometric alterations were evident. The maxillary incisors were significantly retracted, as depicted by the decreased 1.NA and 1-NA values at the end of treatment. Despite the slight mandibular forward movement, the mandibular incisors were significantly protruded, as a consequence of compensatory component of Class II malocclusion treatment, which took place both during the orthopedic and orthodontic phases (1.NB, 1-NB and IMPA) (Table 1). The dentoalveolar results were quite suitable, mainly related to overbite and overjet reduction, as well as to achievement of Class I molar relationship without dental extractions. These results are in agreement with literature regarding the Herbst appliance effects, such as maintenance or reduction of maxillary incisors proclination, associated to maxillary dental arch retraction, as well as mandibular incisors proclination and forward movement of the mandibular teeth,^10^ ^-^ ^15^ which are linked to positive although temporary stimulus of Herbst appliance on mandibular growth, and also on condyle and glenoid fossa forward remodeling.^26^ ^,^ ^32^ ^,^ ^33^

There were no false expectations among orthodontist and parents about an ideal facial improvement, and that was very important in Case 1 treatment. In these terms, is very important to highlight the positive but transitory effect of Herbst appliance on facial profile. As expected,^26^ it was achieved a certain restriction of the forward movement of maxilla during the orthopedic phase, however with minor effect on the mandibular body length. The treatment overall result might be considered positive, mainly due to the decrease of facial convexity, associated to the achievement of spontaneous lip contact in rest position (Fig 6). 

The overall result can be considered satisfactory in Case 2, as well, since both aesthetic and functional goals were achieved, mainly due to patient’s good growth potential during the appropriate stage of skeletal maturation. Therefore, the orthopedic approach reduced the need for extensive tooth movement during the full orthodontic fixed appliances phase. The palatal and mandibular planes were kept on similar inclinations in both pre- and post-treatment phases, witch corroborates the good facial growth pattern of the patient. 

The dentoalveolar compensatory factor, which is inherent to mandibular orthopedic advancement,^8^ ^,^ ^11^ ^,^ ^12^ did not negatively impact the effectiveness of orthopedic treatment. There was great improvement on the reduction of facial profile convexity, as well as on the correction of maxillary and mandibular incisors inclination at the end of treatment. 

Evaluating the final results of both cases, it is reasonable to confirm the evidences pointed out by the literature about Herbst appliance. This device is more effective on the orthopedic treatment of hypodivergent patients, which present a more forward direction of mandibular growth,^8^ ^,^ ^11^ ^,^ ^12^ ^,^ ^32^ as can be highlighted in Case 2. On the other hand, the hyperdivergent patients usually present a limited facial improvement after the same orthopedic approach, with more effect on dentoalveolar structures.^10^ ^-^ ^15^ ^,^ ^33^ It must also be emphasized that the minor mandibular growth response in Case 1 can be attributed to the early skeletal maturation stage of the patient in which orthopedic treatment took place - in other words, before the peak of pubertal growth spurt. 

## CONCLUSION

Considering the results presented in the current paper, it can be concluded that Herbst appliance was efficient in both cases, specially in relation to dentoalveolar effects, which are necessary for Class II correction. The facial and skeletal results, however, were not similar comparing the two cases. In face of high expectations about significant orthopedic results on mandibular growth and its forward movement achieved with Herbst appliance, therefore, two key factors must be considered: the facial growth pattern and the skeletal maturation stage of the patient at treatment onset.
